# Detecting spatio-temporal mortality clusters of European countries by sex and age

**DOI:** 10.1186/s12939-018-0750-z

**Published:** 2018-03-27

**Authors:** Patricia Carracedo, Ana Debón, Adina Iftimi, Francisco Montes

**Affiliations:** 1 Universidad Internacional de Valencia, Área de empresa, c/Pintor Sorolla, Valencia, 21, 46022 Spain; 2 Centro de Gestión de la Calidad y del Cambio, Universitat Politècnica de València, Camino de Vera, s/n, Valencia, 46022 Spain; 3 Departament d’Estadística i I.O., Universitat de València, Valencia, Spain

**Keywords:** Comparative Mortality Figure, Spatial cluster, Local Moran’s Index, Spatial Markov, Europe

## Abstract

**Background:**

Mortality decreased in European Union (EU) countries during the last century. Despite these similar trends, there are still considerable differences in the levels of mortality between Eastern and Western European countries. Sub-group analysis of mortality in Europe for different age and sex groups is common, however to our knowledge a spatio-temporal methodology as in this study has not been applied to detect significant spatial dependence and interaction with time. Thus, the objective of this paper is to quantify the dynamics of mortality in Europe and detect significant clusters of mortality between European countries, applying spatio-temporal methodology. In addition, the joint evolution between the mortality of European countries and their neighbours over time was studied.

**Methods:**

The spatio-temporal methodology used in this study takes into account two factors: time and the geographical location of countries and, consequently, the neighbourhood relationships between them. This methodology was applied to 26 European countries for the period 1990-2012.

**Results:**

Principally, for people older than 64 years two significant clusters were obtained: one of high mortality formed by Eastern European countries and the other of low mortality composed of Western countries. In contrast, for ages below or equal to 64 years only the significant cluster of high mortality formed by Eastern European countries was observed. In addition, the joint evolution between the 26 European countries and their neighbours during the period 1990-2012 was confirmed. For this reason, it can be said that mortality in EU not only depends on differences in the health systems, which are a subject to national discretion, but also on supra-national developments.

**Conclusions:**

This paper proposes statistical tools which provide a clear framework for the successful implementation of development public policies to help the UE meet the challenge of rethinking its social model (Social Security and health care) and make it sustainable in the medium term.

## Background

A first transition phase in mortality in Europe spans from the seventeenth to the early nineteenth century and is characterised by a drop and even disappearance of the mortality crisis caused by infectious diseases (plague, smallpox and typhus). The late nineteenth century was also characterised by a decrease in mortality in most of Europe, mainly due to a drop in diseases like diarrhoea and tuberculosis [36]. This led to a decrease in juvenile mortality, followed by a decline in child mortality. A third period began after World War II and lasted until the 1960s; some authors connect it to the discovery and use of sulphonamide and antibiotics (epidemiologial transition) [11]. Thus, in the early 1960s, European life expectancies converged towards a maximum that Sweden had almost reached by then [26]. Following the mid 1960s, the convergence situation changed due to the Eastern European countries which were governed by Communist regimes. These communist countries were assaulted by health crises which hampered their progress, thereby increasing mortality, while Western European countries began to progress due to new advances in health care, especifically in the treatment of cardiovascular diseases [26]. In 1989 with the collapse of the Berlin wall, life expectancy increased in central and eastern European countries in response to political, social and economic change. The post-1989 increase in life expectancy in these countries has continued [22]. However, Russia and other Soviet countries including Estonia, Latvia and Lithuania have followed a different path. The collapse of the Soviet Union mainly affected Russia decreasing its life expectancy. By then, the Baltic States were independent countries, which compared to other countries emerging from the former Soviet Union stand out with respect to democratic rights, economic reforms and improved living conditions [39]. At the end of 2008, the Russian Ministry of Health proposed a set of ambitious targets for health improvement to increase life expectancy in the future [22]. Therefore, European countries have been undergoing a divergent situation between the West and the East.

It is well known that eastern European countries have a mortality which is higher than other countries. For example, Vaupel et al. studied 40 developed countries and showed that eastern European countries have a lower life expectancy than the other countries considered [43]. In line with previous studies such as Meslé, in most central/western European countries, life expectancy was increasing mainly due to a decline in cardiovascular mortality and economic growth [25]. In addition, other authors such as Mackenbach, Karanikolos and McKee [24] conclude that mortality trends in Eastern Europe are affected by economic and health-care issues and unsuccessful implementation of effective health-care policies. Hatzopoulos and Haberman [18] classified 35 countries for the time period 1960-2006 into western and eastern clusters. The eastern cluster had a sub-cluster consisting mainly of Russia, Ukraine and Belarus. For these countries, the mortality dynamics are distinguishable from the rest of the eastern cluster countries (Estonia, Hungary, Lithuania, Latvia, Bulgaria), and their life expectancies decreased after the 1990’s. More recently, Debón et al. [12], through clustering method, concluded that there are clear differences in mortality between the east and west of the EU, with a significant disadvantage for Eastern Europe, and especially for males in Baltic countries.

Many of the studies mentioned above consider the well-known life expectancy, but none of them uses a spatio-temporal methodology to detect the dependence on mortality between European countries over time.

This study is motivated by interest in the inequalities between the health systems of different countries in the EU [38] applying spatio-temporal methodology which that as far as we know has not been used in this field. This methodology takes into account: time, the neighbourhood relationships between the European countries and their interaction.

For this reason, the main objective of this paper is to quantify the dynamics of mortality in Europe and detect significant clusters of mortality between European countries, applying spatio-temporal methodology. In addition, the temporal evolution of the mortality of European countries and their neighbours jointly was studied.

This paper is structured as follows. “Methods” section starts by describing the basis of the data from selected countries. This section ends with an exposition of the mortality measures and spatio-temporal methodology used to detect significant differences in mortality between countries: Comparative Mortality Figure, Global Moran’s Index, Local Moran’s Index and Spatial Markov. “Results” section presents the main results of this spatio-temporal study and finally, in “Conclusions” section, the most important conclusions of the study are shown.

## Methods

### Data

In order to detect significant differences in mortality between European countries, the unit of analysis is each country. The data was downloaded from the Human Mortality Database (HMD) [17]. For this reason, this study takes into account mortality data for 26 European countries: Austria, Belgium, Belarus, The Czech Republic, Denmark, Estonia, Finland, France, Germany, Hungary, Ireland, Italy, Latvia, Lithuania, Luxembourg, The Netherlands, Norway, Poland, Portugal, Slovakia, Slovenia, Spain, Sweden, Switzerland, The United Kingdom and Ukraine. These countries have a maximum common time range from 1990 to 2012, for the age groups from 0 to 110+ for both men and women. In short, we have a total of 26 countries and 23 years.

Mortality data for countries such as Greece and Bulgaria are not considered as there is no information corresponding to their neighbours such as Romania, Albania and Macedonia and, in addition, it was not considered a good idea to force the neighbourhood structure. However, taking into account proximity and socio-economic reasons, the neighbourhood structure was modified by considering Sweden and Norway neighbours of Denmark and France a neighbour of The United Kingdom.

These mortality data were downloaded from the HMD using the new R package called HMDHFDplus by Riffe [35], specifically the readHMDweb function, and not the usual package called demography by Hyndman et al. [16] with the hmd.mx function. Thanks to the readHMDweb function, mortality data was downloaded in an easier way to manipulate in R [32] and the observed deaths were directly obtained. In this way, for each country a data.frame object with 4 variables (year, age, number of deaths and population) and 2553 observations (23 years ×111 ages) was obtained. In contrast, with the function hmd.mx for each country, a list object with 6 elements (country, type of data, year, age, death rate and population) is obtained. The elements age and year are vectors and the death rate and the population are lists. In this case, the observed deaths are obtained indirectly by multiplying the population by the death rate.

In order to analyze the behaviour for important ages, the same three age groups that the International Monetary Fund considers for calculation of the dependency ratio were taken into account [23]. The old dependency ratio is defined as the number of people older than 64 divided by the number of individuals aged between 15 and 64, which is crucial for pay-as-you-go pension systems. Therefore, the studied age groups for both sexes were: between 0 and 14 years of age (*g*_1_), between 15 and 64 years of age (*g*_2_) and between 65 and 110+ years of age (*g*_3_).

Statistical analysis was performed using the free software R and some specific R-packages: devtools [44], HMDHFDplus [35], maptools [7], spdep [4, 9], GeoXp [21], rgdal [6], Gmisc [14] and RColorBrewer [29].

### Statistics

#### Statistics for Comparison of Mortality

To compare crude mortality rates from different geographical areas, standardization of these rates is required. Standardization permits comparison of sub-group free population size [20]. In addition, it is important to specify the standard population used in the standardization because this choice can affect the results [30] although it is an arbitrary decision. In this study, the standard population is the set of European countries for both sexes as the deaths and the population of total of Europe are not observed directly [20].

There are two methods of standardization: direct and indirect, as shown by Fleiss, Levin and Paik [13]. The direct method produces the Comparative Mortality Figure (CMF) and the indirect method the Standardised Mortality Ratio (SMR). Both ratios are defined as follows.

The SMR is well-known in epidemiology [15]. In this paper, it is obtained for each age group *g*

1 $$\begin{array}{@{}rcl@{}} SMR_{i,g,t,s}= \frac{O_{i,g,t,s}}{E_{i,g,t,s}}& & \quad \text{for g} \in \{g_{1}, g_{2},g_{3}\},\\ && \quad \mathrm{i} \in \{{1,\ldots,N}\}, \mathrm{t} \in \{1,\ldots,T\}\\ && \quad \text{and s} \in \{male, female\} \end{array} $$

where *O*_*i*,*g*,*t*,*s*_ represents the number of observed deaths for each European country *i*, age group *g*, year *t* and sex *s*, and *E*_*i*,*g*,*t*,*s*_ the corresponding expected deaths under the hypothesis that each country has the same mortality as the set of European countries. If *x* represents the age of death, the *O*_*i*,*g*,*t*,*s*_ are obtained by means of

2 $$ O_{i,g,t,s}= \sum\limits_{x \in g}^{}d_{i,x,t,s},  $$

where *d*_*i*,*x*,*t*,*s*_ represents the number of deaths. The *E*_*i*,*g*,*t*,*s*_ are obtained as follows

3 $$ E_{i,g,t,s}= \sum\limits_{x \in g}^{}{m_{x,t}p_{i,x,t,s},}  $$

where *p*_*i*,*x*,*t*,*s*_ is the studied population and *m*_*x*,*t*_ is the death rate in the set of European countries for both sexes

4 $$ m_{x,t}= \frac{{\sum\limits_{i=1}^{N} \sum\limits_{s}^{}} d_{i,x,t,s}}{\sum\limits_{i=1}^{N}\sum\limits_{s}^{}p_{i,x,t,s}}.  $$

If the SMR is greater than 1, there is a higher number of deaths than expected, in this case there are “excess deaths” in the European country *i*.

The CMF is expressed as

5 $$\begin{array}{@{}rcl@{}} CMF_{i,g,t,s}= \frac{E_{i,g,t,s}}{O_{g,t}}& & \quad \text{for g} \in \{g_{1}, g_{2},g_{3}\},\\ && \quad \mathrm{i} \in \{{1,\ldots,N}\}, \mathrm{t} \in \{1,\ldots,T\}\\ && \quad \text{and s} \in \{{male, female\}} \end{array} $$

where *E*_*i*,*g*,*t*,*s*_ is defined as the corresponding expected deaths for each European country *i*, age group *g*, year *t* and sex *s* and *O*_*g*,*t*_ represents the number of observed deaths in the set of European countries. If *x* represents the age of death, the *E*_*i*,*g*,*t*,*s*_ are obtained as follows

6 $$\begin{array}{@{}rcl@{}} E_{i,g,t,s}=\sum\limits_{x \in g}^{} {m_{i,x,t,s}P_{x,t}}, \end{array} $$

where *m*_*i*,*x*,*t*,*s*_ is the death rate and *P*_*x*,*t*_ the standard population. The death rate of the study population are obtained as

7 $$ m_{i,x,t,s}= \frac{d_{i,x,t,s}}{p_{i,x,t,s}},  $$

where *d*_*i*,*x*,*t*,*s*_ and *p*_*i*,*x*,*t*,*s*_ are the number of deaths and the study population respectively. *P*_*x*,*t*_ is the standard population which is the population of the set of countries, and it can be obtained by expression (8),

8 $$ P_{x,t}= \sum\limits_{i=1}^{N}\sum\limits_{s}^{} p_{i,x,t,s},  $$

The *O*_*g*,*t*_ for both sexes are obtained by means of

9 $$ O_{g,t}= \sum\limits_{i=1}^{N}\sum\limits_{x \in g}^{}\sum\limits_{s}^{} d_{i,x,t,s}.  $$

If the CMF is greater than 1, it represents an unfavourable mortality experience, in the same way as with the SMR.

In order to compare the mortality over time for different countries by sex, the CMF has been used in this study for two reasons. First, the same denominator applies in the calculation for each country which permits comparison of the mortality experience by sex for different countries and second, the age-specific mortality rates are available for each country [20] required in expression (6) but not in expression (3).

#### Global Moran’s Index

The Global Moran’s I [27, 28] is a summary measure of the intensity of the spatial autocorrelation between territorial units. It is defined by

10 $$ {\begin{aligned} G&M_{g,t,s}=\\ &\!\!\!\frac{N\sum\limits_{i}^{}\sum\limits_{j}^{}\omega_{ij}\left(CMF_{i,g,t,s}-\overline{CMF}_{g,t,s}\right)\!\!\left(CMF_{g,j,t,s}-\overline{CMF}_{g,t,s}\right)}{\sum\limits_{i}^{}\!\sum\limits_{j}^{}\omega_{ij}\sum\limits_{i}^{}\left(CMF_{i,g,t,s}-\overline{CMF}_{g,t,s}\right)^{2}}. \end{aligned}}  $$

In this formula, *CMF*_*i*,*g*,*t*,*s*_ was defined in expression (5), $\overline {CMF}_{g,t,s}$ is the mean of the CMF in all the countries for each age group *g*, year *t* and sex *s* and *W*=(*ω*_*ij*_) is the matrix of spatial weights.

Regarding *W*, spatial data exploration requires the setting of a neighbourhood structure between geographic units that represents the configuration of the analyzed territory. The units are usually administrative divisions established in a given territory.

The neighbourhood structure depends on the criteria used to define the concept of neighbour. Neighbours are defined as the administrative units that share a border, an approach that seems best suited to an irregular (lattice) such as the one of the different countries in Europe; *W* has the following form:

11 $$\begin{array}{@{}rcl@{}}{} \omega_{ii}&=&0, \quad \hbox {\(i=1,..,N\);}  \\ \omega_{ij}&=&\frac{1}{n_{i}}, \, \text{ if } j\in V(i), with n_{i}=\#V(i); \\ \omega_{ij}&=&0, \quad \text{if } j\notin V(i),  \end{array} $$

where *i* and *j* represent whichever two of the *N* countries, *n*_*i*_ number of neighbours of *i* and *V*(*i*) the set of neighbours of country *i*. With this structure, no country is its own neighbour, and the values in each row sum to unity because the weights *ω*_*ij*_ are standardised. Only the first order neighbourhood structure has been used in this study. For other neighbouring structures, see Cliff and Ord [10].

It can be seen that *GM*_*g*,*t*,*s*_ in expression (10) is the slope of the least squares regression line that best fits the points between the *CMF*_*i*,*g*,*t*,*s*_ and its neighbours average. Negative values of this index indicate that there is negative spatial autocorrelation, i.e. the CMF of countries and their neighbours varies in a different direction, and positive values indicate that there is positive spatial autocorrelation, i.e. the CMF of countries and their neighbours goes in the same direction. Values of this index near zero indicate the absence of a spatial autocorrelation between the 26 European countries, meaning a random spatial pattern.

#### Test for spatial autocorrelation

Moran’s test (M) for spatial autocorrelation was calculated to determine whether the data show spatial autocorrelation. Cliff and Ord [10] derive the distribution of *GM*_*g*,*t*,*s*_ to test the null hypothesis of no spatial correlation (*H*_0_:*GM*_*g*,*t*,*s*_=0). Moran’s I has largely been used over the last decades, but as Wilcox [45] argued, this may be very sensitive to even a single value, which could change the slope of the regression line. To assess the robustness of the estimated Moran’s I, the Monte-Carlo test (MC) and a Robust Index (RI) proposed by Wilcox [45] were computed. The Monte-Carlo test (MC) uses random permutations of *CMF*_*i*,*g*,*t*,*s*_ for the given the matrix of spatial weights, to establish the rank of the observed statistic in relation to the 999 simulated values. The Monte Carlo test overcomes the disadvantage of the problem of asymptotic normality. The result of the contrast contains the following components: the value of the observed Moran’s I, the rank of the observed Moran’s I and the *p*-value of the Monte-Carlo test [10]. The robust statistic is a modification of Wilcox’s Percentage Bend Correlation [45]. This measure of association falls under the M-Measures type of correlation and is implemented in R in http://www.colby.edu/~mgimond/R/RobustI.R.

#### Local Moran’s Index

Anselin introduced the concept of Local Indicators of Spatial Association (LISA), a local version of Global Moran Index for the identication of signicant local spatial association [2]. It is defined by

12 $$\begin{array}{*{20}l} {}L&M_{i,g,t,s}=\\ {}&\frac{(CMF_{i,g,t,s}-\overline{CMF}_{g,t,s})}{S^{2}(CMF_{g,t,s})} \sum\limits_{i}^{}\sum\limits_{j}^{}\omega_{ij}(CMF_{g,j,t,s}-\overline{CMF}_{g,t,s}) \end{array} $$

where *S*^2^(*CMF*_*g*,*t*,*s*_) is the variance of the CMF in all the countries for each age group *g*, year *t* and sex *s*. In the notation which has become usual in this context, L and H denote, respectively, CMF values of a country that are Lower (L) or Higher (H) than the global mean of countries’ CMF. Similarly, L and H denote, respectively, mean CMF of neighbours that are Lower (L) or Higher (H) than the global mean of neighbours averages. Therefore, each observation could be placed into one of four states, where the first letter indicates the CMF values of a country and the second to the mean of its neighbours as summarized in Table 1.

**Table 1:** LISA Classifications for each country and its neighbourhood

State	Country’CMF	Neighbours’ $\overline {CMF}$
1=LL	Below average	Below average
2=LH	Below average	Above average
3=HL	Above average	Below average
4=HH	Above average	Above average

LL.- Countries with low values of CMF surrounded by neighbours also with low values of CMF LH.- Countries with low values of CMF surrounded by neighbours with high values of CMF HL.- Countries with high values of CMF surrounded by neighbours with low values of CMF HH.- Countries with high values of CMF surrounded by neighbours also with high values of CMF

When the Local Moran’s Index is significant, it indicates two types of associations:

- *Spatial clusters*.- A positive *LM*_*i*,*g*,*t*,*s*_ indicates a cluster of countries with high values of CMF surrounded by neighbours also with high values of CMF, denoted by HH, or spatial cluster of countries with low values of CMF surrounded by neighbours also with low values of CMF denoted by LL.
- *Outlier associations*.- A negative *LM*_*i*,*g*,*t*,*s*_ indicates spatial outlier countries with high values of CMF surrounded by neighbours with low values of CMF, denoted by HL or spatial outlier countries with low values of CMF surrounded by neighbours with high values of CMF, denoted by LH.

To obtain the *p*-values of the local Moran index based on the number of neighbours in each country, the Bonferroni correction was used. The Bonferroni adjustment is a multiple comparison test, which divides the level of significance *α* by the average number of neighbours in each test. If the adjusted *p*-value is less than 0.05 the country is classified as a significant association [2].

#### Spatial Markov

The above techniques allow us to study the spatial autocorrelation in a static manner. *GM*_*g*,*t*,*s*_ and *LM*_*i*,*g*,*t*,*s*_ are obtained for each year and therefore do not allow for a investigation of their joint spatio-temporal evolution. In order to determine whether there is interaction between space and time, Rey [33, 34] extends the process components introduced by Quah [31] to study the dynamic evolution of regional income. Following that study, the different European countries and the average *CMF* value of their neighbourhood can be classified into 4 states described in Table 1. The observed joint transition probability matrix $P(country'CMF, neighbours'\overline {CMF})$ of dimension 4×4 is formed by the transition probabilities each 4 states from 1990 to 2012,

$${}P(country'CMF, neighbours'\overline{CMF}) = \left(\begin{array}{cccc} p_{11} & p_{12} & p_{13} & p_{14}  \\ p_{21} & p_{22}& p_{23} & p_{24}  \\ p_{31} & p_{32}& p_{33} & p_{34} \\ p_{41} & p_{42} & p_{43} & p_{44} \\ \end{array} \right) $$

where *p*_*lm*_ is the joint probability of a country and its neighbourhood moving from state *l* to state *m* from 1990 to 2012, where *l*={1,2,3,4} and *m*={1,2,3,4} are ordered as Table 1.

In order to estimate *p*_*lm*_ for a stationary chain, it is necessary to obtain the observed number of transitions *ν*_*lm*_ from *l* to *m* from 1990-2012 in a matrix of dimension 4×4. The estimate of *p*_*lm*_ is the *ν*_*lm*_ divided by the sum of number of transitions in the row. The *ν*_*lm*_ is obtained as the sum of observed number of transitions from *l* to *m* in all 22 pair of years of Table 2.

**Table 2:** Observed number of transitions from state for a country and its neighbourhood to the next period

Country ∖ Pair of years	(1990,1991)	(1991,1992)	(1992,1993)	(1993,1994)	(1994,1995)	(1995,1996)	(1996,1997)	(1997,1998)	(1998,1999)	(1999,2000)	(2000,2001)
Austria	(1,1)	(1,1)	(1,1)	(1,1)	(1,1)	(1,1)	(1,1)	(1,1)	(1,1)	(1,1)	(1,1)
Belgium	(1,1)	(1,1)	(1,1)	(1,1)	(1,1)	(1,1)	(1,1)	(1,1)	(1,1)	(1,1)	(1,1)
Belarus	(4,4)	(4,4)	(4,4)	(4,4)	(4,4)	(4,4)	(4,4)	(4,4)	(4,4)	(4,4)	(4,4)
Switzerland	(1,1)	(1,1)	(1,1)	(1,1)	(1,1)	(1,1)	(1,1)	(1,1)	(1,1)	(1,1)	(1,1)
Czech Republic	(4,4)	(4,2)	(2,2)	(2,2)	(2,1)	(1,2)	(2,1)	(1,1)	(1,1)	(1,1)	(1,1)
Germany	(1,1)	(1,1)	(1,1)	(1,1)	(1,1)	(1,1)	(1,1)	(1,1)	(1,1)	(1,1)	(1,1)
Denmark	(1,1)	(1,1)	(1,1)	(1,1)	(1,1)	(1,1)	(1,1)	(1,1)	(1,1)	(1,1)	(1,1)
Estonia	(4,4)	(4,4)	(4,4)	(4,4)	(4,4)	(4,4)	(4,4)	(4,4)	(4,4)	(4,4)	(4,4)
Spain	(2,2)	(2,1)	(1,1)	(1,1)	(1,1)	(1,1)	(1,1)	(1,1)	(1,1)	(1,1)	(1,1)
Finland	(1,1)	(1,1)	(1,1)	(1,1)	(1,1)	(1,1)	(1,1)	(1,1)	(1,1)	(1,1)	(1,1)
France	(1,1)	(1,1)	(1,1)	(1,1)	(1,1)	(1,1)	(1,1)	(1,1)	(1,1)	(1,1)	(1,1)
Hungary	(4,4)	(4,4)	(4,4)	(4,4)	(4,4)	(4,4)	(4,4)	(4,4)	(4,4)	(4,4)	(4,4)
Ireland	(1,1)	(1,1)	(1,1)	(1,1)	(1,1)	(1,1)	(1,1)	(1,1)	(1,1)	(1,1)	(1,1)
Italy	(1,1)	(1,1)	(1,1)	(1,1)	(1,1)	(1,1)	(1,1)	(1,1)	(1,1)	(1,1)	(1,1)
Lithuania	(4,4)	(4,4)	(4,4)	(4,4)	(4,4)	(4,4)	(4,4)	(4,4)	(4,4)	(4,4)	(4,4)
Luxembourg	(1,1)	(1,1)	(1,3)	(3,1)	(1,1)	(1,1)	(1,1)	(1,1)	(1,1)	(1,1)	(1,1)
Latvia	(4,4)	(4,4)	(4,4)	(4,4)	(4,4)	(4,4)	(4,4)	(4,4)	(4,4)	(4,4)	(4,4)
Netherlands	(1,1)	(1,1)	(1,1)	(1,1)	(1,1)	(1,1)	(1,1)	(1,1)	(1,1)	(1,1)	(1,1)
Norway	(1,1)	(1,1)	(1,1)	(1,1)	(1,1)	(1,1)	(1,1)	(1,1)	(1,1)	(1,1)	(1,1)
Poland	(4,4)	(4,4)	(4,4)	(4,4)	(4,4)	(4,4)	(4,4)	(4,4)	(4,4)	(4,4)	(4,4)
Portugal	(3,3)	(3,3)	(3,3)	(3,3)	(3,3)	(3,3)	(3,3)	(3,3)	(3,3)	(3,3)	(3,1)
Sweden	(1,1)	(1,1)	(1,1)	(1,1)	(1,1)	(1,1)	(1,1)	(1,1)	(1,1)	(1,1)	(1,1)
Slovenia	(1,2)	(2,1)	(1,1)	(1,1)	(1,1)	(1,1)	(1,1)	(1,1)	(1,1)	(1,1)	(1,1)
Slovakia	(4,4)	(4,4)	(4,4)	(4,4)	(4,4)	(4,4)	(4,4)	(4,4)	(4,4)	(4,4)	(4,4)
Ukraine	(4,4)	(4,4)	(4,4)	(4,4)	(4,4)	(4,4)	(4,4)	(4,4)	(4,4)	(4,4)	(4,4)
Uk	(1,1)	(1,1)	(1,1)	(1,1)	(1,1)	(1,1)	(1,1)	(1,1)	(1,1)	(1,1)	(1,1)
Austria	(1,1)	(1,1)	(1,1)	(1,1)	(1,1)	(1,1)	(1,1)	(1,1)	(1,1)	(1,1)	(1,1)
Belgium	(1,1)	(1,1)	(1,1)	(1,1)	(1,1)	(1,1)	(1,1)	(1,1)	(1,1)	(1,1)	(1,3)
Belarus	(4,4)	(4,4)	(4,4)	(4,4)	(4,4)	(4,4)	(4,4)	(4,4)	(4,4)	(4,4)	(4,4)
Switzerland	(1,1)	(1,1)	(1,1)	(1,1)	(1,1)	(1,1)	(1,1)	(1,1)	(1,1)	(1,1)	(1,1)
Czech Republic	(1,1)	(1,2)	(2,2)	(2,2)	(2,2)	(2,2)	(2,2)	(2,2)	(2,2)	(2,2)	(2,2)
Germany	(1,1)	(1,1)	(1,1)	(1,1)	(1,1)	(1,1)	(1,1)	(1,1)	(1,1)	(1,1)	(1,1)
Denmark	(1,1)	(1,1)	(1,1)	(1,1)	(1,1)	(1,1)	(1,1)	(1,1)	(1,1)	(1,1)	(1,1)
Estonia	(4,4)	(4,4)	(4,4)	(4,4)	(4,4)	(4,4)	(4,4)	(4,2)	(2,4)	(4,2)	(2,2)
Spain	(1,1)	(1,1)	(1,1)	(1,1)	(1,1)	(1,1)	(1,1)	(1,1)	(1,1)	(1,1)	(1,1)
Finland	(1,1)	(1,1)	(1,1)	(1,1)	(1,1)	(1,1)	(1,1)	(1,1)	(1,1)	(1,1)	(1,1)
France	(1,1)	(1,1)	(1,1)	(1,1)	(1,1)	(1,1)	(1,1)	(1,1)	(1,1)	(1,1)	(1,1)
Hungary	(4,4)	(4,4)	(4,4)	(4,4)	(4,4)	(4,4)	(4,4)	(4,4)	(4,4)	(4,4)	(4,4)
Ireland	(1,1)	(1,1)	(1,1)	(1,1)	(1,1)	(1,2)	(2,1)	(1,1)	(1,2)	(2,2)	(2,2)
Italy	(1,1)	(1,1)	(1,1)	(1,1)	(1,1)	(1,1)	(1,1)	(1,1)	(1,1)	(1,1)	(1,1)
Lithuania	(4,4)	(4,4)	(4,4)	(4,4)	(4,4)	(4,4)	(4,4)	(4,4)	(4,4)	(4,4)	(4,4)
Luxembourg	(1,3)	(3,1)	(1,1)	(1,1)	(1,1)	(1,1)	(1,1)	(1,1)	(1,1)	(1,1)	(1,1)
Latvia	(4,4)	(4,4)	(4,4)	(4,4)	(4,4)	(4,4)	(4,4)	(4,4)	(4,4)	(4,4)	(4,4)
Netherlands	(1,1)	(1,1)	(1,1)	(1,1)	(1,1)	(1,1)	(1,1)	(1,1)	(1,1)	(1,1)	(1,1)
Norway	(1,1)	(1,1)	(1,1)	(1,1)	(1,1)	(1,1)	(1,1)	(1,1)	(1,1)	(1,1)	(1,1)
Poland	(4,4)	(4,4)	(4,4)	(4,4)	(4,4)	(4,4)	(4,4)	(4,4)	(4,4)	(4,4)	(4,4)
Portugal	(1,1)	(1,1)	(1,1)	(1,1)	(1,1)	(1,1)	(1,1)	(1,1)	(1,1)	(1,1)	(1,1)
Sweden	(1,1)	(1,1)	(1,1)	(1,1)	(1,1)	(1,1)	(1,1)	(1,1)	(1,1)	(1,1)	(1,1)
Slovenia	(1,1)	(1,1)	(1,1)	(1,1)	(1,1)	(1,1)	(1,1)	(1,1)	(1,1)	(1,1)	(1,1)
Slovakia	(4,4)	(4,4)	(4,4)	(4,4)	(4,4)	(4,4)	(4,4)	(4,4)	(4,4)	(4,4)	(4,4)
Ukraine	(4,4)	(4,4)	(4,4)	(4,4)	(4,4)	(4,4)	(4,4)	(4,4)	(4,4)	(4,4)	(4,4)
Uk	(1,1)	(1,1)	(1,1)	(1,1)	(1,1)	(1,3)	(3,1)	(1,1)	(1,3)	(3,3)	(3,3)

For example, Belgium and its neighbourhood stayed in the same state 1 from 1990 to 2011, but changed from state 1 in 2011 to 3 in 2012.

From the mortality data observed from 1990 to 2012, the transition probability matrices *P*(*country*^′^*CMF*) and $P(neighbours'\overline {CMF})$ can be estimated separately, both with dimensions of 2 ×2. Under the hypothesis of independence or lack of spatial dynamics,

$\widehat {P(country'CMF,neighbours'\overline {CMF})}$=$\widehat {P(country'CMF)} \otimes \widehat {P(neighbours'\overline {CMF})}$, where ⊗ represents the Kronecker product and the estimated joint transition probability matrix with dimension of 4 ×4 is

$\widehat {P(country'CMF,neighbours'\overline {CMF})}=\widehat {p_{lm}}$.

Estimates of the corresponding observed and estimated joint transition probability matrices are compared, and a formal test *χ*^2^[1] permits testing of the equality of both matrices. Under the null hypotesis $p_{lm} = \widehat {p_{lm}}$,

13 $$ \sum\limits_{m=1}^{4}\nu_{l}^{*}\frac{(p_{lm}-{\widehat{p_{lm}})}^{2}}{\widehat{p_{lm}}}  $$

has an asymptotic *χ*^2^ distribution with (4−1) degrees of freedom where $\phantom {\dot {i}\!}\nu _{l}^{*}=\sum \limits _{m=1}^{4}\nu _{lm}$. Since the variables $\nu _{l}^{*} (p_{lm}-{\widehat {p_{lm}}})^{2}$ for different *l* are asymptotically independent, the forms of Eq. (13) for different *l* are asymptotically independent, and hence can be added to obtain other *χ*^2^ variables [1]. As our aim is to have a test for all *p*_*lm*_ where *l*,*m*={1,2,3,4}, it can be obtain by adding over all *l* in Eq. (13), resulting in a *χ*^2^ variable with 4×(4−1) degrees of freedom [1, 19]. Its rejection means that the Markov chain associated with country-environment co-evolution is not separable, meaning that spatio-temporal interaction exists.

## Results

For the sake of brevity, only the most representative results and maps are shown here. Readers interested in the results and maps for all the years can request them from the authors.

To quantify and study relevant facts of the annual evolution of the CMF in Europe by sex and age group for the period 1990-2012, Tables 3 and 8 were produced. Tables 3 and 8 include in columns: the CMF of European countries for the period 1990-2012 every three years, the value of the mean, standard deviation (SD) and relative variation in percentage (RV %) of CMF for the whole period for each country. In addition, Tables 3 and 8 show in rows: name of the country, mean of western countries, eastern countries, countries of the former Union of Soviet Socialist Republics (USSR) and the other former communist countries. Tables 3, 4 and 5 include the CMF of European countries for men and Tables 6, 7 and 8 the same measure for women.

**Table 3:** Behaviour of the CMF in Europe countries for men between 0 and 14 ages over the period 1990-2012

East/West	Country	1990	1993	1996	1999	2002	2005	2008	2011	Mean	SD	RV%
West	Austria	0.88	0.86	0.79	0.76	0.87	0.90	0.93	1.03	0.88	0.07	-3.11
West	Belgium	0.95	1.01	0.87	0.94	0.96	0.93	0.94	0.98	0.95	0.06	17.95
West	Denmark	0.95	0.74	0.87	0.85	0.85	0.95	0.97	0.80	0.87	0.08	-14.51
West	Finland	0.68	0.65	0.68	0.73	0.67	0.91	0.78	0.72	0.71	0.07	-3.28
West	France	0.88	0.88	0.82	0.84	0.87	0.85	0.88	0.89	0.88	0.04	6.66
West	Germany	0.89	0.80	0.80	0.83	0.81	0.87	0.85	0.95	0.85	0.05	1.70
West	Ireland	0.93	0.83	0.94	1.11	1.04	0.77	1.03	0.91	0.95	0.11	-11.78
West	Italy	0.88	0.91	0.90	0.86	0.92	0.80	0.84	0.82	0.85	0.06	-11.18
West	Luxembourg	0.81	1.17	0.88	0.98	1.49	0.64	0.61	1.08	0.87	0.24	-30.03
West	Netherlands	0.88	0.85	0.87	0.92	1.00	1.01	0.86	0.97	0.92	0.07	15.64
West	Norway	0.91	0.73	0.72	0.80	0.75	0.79	0.76	0.73	0.76	0.05	-18.95
West	Portugal	1.42	1.38	1.30	1.20	1.19	0.90	0.85	0.95	1.13	0.22	-39.27
West	Spain	0.91	0.89	0.92	0.86	0.90	0.90	0.86	0.83	0.87	0.04	-10.14
West	Sweden	0.71	0.66	0.56	0.67	0.68	0.65	0.59	0.61	0.66	0.05	6.12
West	Switzerland	0.82	0.74	0.79	0.89	0.89	0.98	0.90	0.99	0.87	0.07	10.06
West	The United Kingdom	0.92	0.83	0.90	0.98	1.03	1.08	1.09	1.14	0.99	0.10	18.90
Mean of western countries		0.90	0.87	0.85	0.89	0.93	0.87	0.86	0.90	0.88	0.09	-5.40
East	Belarus ^*a*^	1.48	1.72	1.98	2.32	1.96	1.94	1.42	1.39	1.78	0.28	-13.41
East	The Czech Republic	1.22	1.13	0.91	0.89	0.95	0.89	0.83	0.78	0.96	0.13	-32.25
East	Estonia ^*a*^	1.77	1.92	1.75	1.85	1.62	1.38	1.45	0.94	1.62	0.39	-47.24
East	Hungary	1.56	1.45	1.38	1.48	1.39	1.43	1.33	1.21	1.41	0.13	-21.71
East	Latvia ^*a*^	1.98	2.20	2.22	2.04	2.31	2.05	1.94	1.86	2.12	0.25	-14.48
East	Lithuania ^*a*^	1.44	1.95	1.54	1.71	1.76	1.95	1.65	1.52	1.72	0.20	-11.60
East	Poland	1.93	1.78	1.65	1.47	1.40	1.37	1.35	1.21	1.49	0.21	-35.25
East	Slovakia	1.33	1.34	1.44	1.49	1.34	1.60	1.55	1.50	1.48	0.09	16.76
East	Slovenia	1.03	0.92	0.88	0.92	0.95	0.93	0.73	0.88	0.86	0.14	-47.75
East	Ukraine ^*a*^	1.70	2.15	2.39	2.49	2.71	2.60	2.63	2.54	2.41	0.28	45.85
Mean of eastern countries		1.55	1.66	1.61	1.67	1.64	1.61	1.49	1.38	1.59	0.21	-15.49
Mean countries of the former USSR		1.68	1.99	1.98	2.08	2.07	1.98	1.82	1.65	1.93	0.29	-8.51
Mean of the other former communist countries		1.42	1.32	1.25	1.25	1.21	1.24	1.16	1.12	1.24	0.15	-23.76

^a^countries of the former USSR

**Table 4:** Behaviour of the CMF in Europe countries for men between 15 and 64 ages over the period 1990-2012

East/West	Country	1990	1993	1996	1999	2002	2005	2008	2011	Mean	SD	RV%
West	Austria	1.23	1.17	1.11	1.07	1.03	1.00	0.97	1.06	1.08	0.07	-14.18
West	Belgium	1.09	1.05	1.03	1.08	1.07	1.04	1.08	1.09	1.06	0.02	1.68
West	Denmark	1.23	1.16	1.14	1.09	1.09	1.02	1.04	1.04	1.11	0.05	-16.39
West	Finland	1.44	1.25	1.19	1.23	1.15	1.24	1.22	1.21	1.23	0.07	-18.75
West	France	1.27	1.22	1.16	1.14	1.15	1.11	1.10	1.17	1.16	0.04	-7.64
West	Germany	1.26	1.18	1.13	1.07	1.05	1.01	1.01	1.09	1.09	0.07	-13.16
West	Ireland	1.12	1.06	1.02	1.05	1.00	0.85	0.87	0.92	0.98	0.08	-17.73
West	Italy	1.04	0.95	0.91	0.88	0.83	0.77	0.79	0.79	0.86	0.09	-23.17
West	Luxembourg	1.21	1.15	1.18	1.05	1.10	1.01	0.87	0.96	1.08	0.10	-18.19
West	Netherlands	0.94	0.92	0.87	0.88	0.86	0.79	0.77	0.78	0.85	0.05	-13.96
West	Norway	1.01	0.93	0.85	0.88	0.87	0.80	0.79	0.84	0.87	0.06	-17.84
West	Portugal	1.34	1.32	1.32	1.30	1.25	1.22	1.16	1.22	1.26	0.05	-9.29
West	Spain	1.09	1.05	1.03	1.00	1.00	0.96	0.92	0.93	1.00	0.05	-14.52
West	Sweden	0.89	0.81	0.76	0.76	0.75	0.73	0.73	0.75	0.77	0.05	-15.46
West	Switzerland	0.99	0.91	0.84	0.82	0.80	0.76	0.72	0.74	0.82	0.08	-23.57
West	The United Kingdom	1.06	0.99	0.95	0.96	0.94	0.90	0.91	0.92	0.95	0.04	-13.91
Mean of western countries		1.14	1.07	1.03	1.01	0.99	0.95	0.94	0.97	1.01	0.06	-14.62
East	Belarus ^*a*^	2.24	2.76	3.03	3.47	3.77	3.83	3.64	4.10	3.34	0.53	56.40
East	The Czech Republic	1.90	1.63	1.57	1.56	1.52	1.47	1.45	1.50	1.56	0.11	-22.52
East	Estonia ^*a*^	2.45	2.97	2.74	2.83	2.88	2.62	2.47	2.26	2.72	0.30	-6.34
East	Hungary	2.46	2.65	2.42	2.56	2.37	2.45	2.37	2.43	2.46	0.09	-5.52
East	Latvia ^*a*^	2.56	3.46	3.02	3.02	3.09	3.23	3.01	2.83	3.09	0.32	12.32
East	Lithuania ^*a*^	2.25	2.88	2.76	2.56	2.77	3.22	3.33	3.12	2.89	0.35	38.40
East	Poland	2.11	1.98	1.94	2.04	1.86	1.93	2.01	2.04	2.00	0.08	-2.21
East	Slovakia	2.20	1.95	1.84	2.00	1.97	1.95	1.97	1.97	1.97	0.08	-11.88
East	Slovenia	1.63	1.70	1.47	1.50	1.47	1.31	1.28	1.23	1.46	0.14	-22.58
East	Ukraine ^*a*^	2.29	2.75	3.33	3.32	3.66	4.12	4.18	3.44	3.41	0.53	52.69
Mean of eastern countries		2.21	2.47	2.41	2.49	2.54	2.61	2.57	2.49	2.49	0.25	10.21
Mean countries of the former USSR		2.36	2.96	2.98	3.04	3.24	3.40	3.33	3.15	3.09	0.42	29.63
Mean of the other former communist countries		2.06	1.98	1.85	1.93	1.84	1.82	1.82	1.83	1.89	0.10	-12.04

^a^countries of the former USSR

**Table 5:** Behaviour of the CMF in Europe countries for men between 65 and 110+ ages over the period 1990-2012

East/West	Country	1990	1993	1996	1999	2002	2005	2008	2011	Mean	SD	RV%
West	Austria	1.28	1.26	1.25	1.22	1.20	1.17	1.17	1.19	1.22	0.04	-7.06
West	Belgium	1.29	1.29	1.27	1.26	1.28	1.24	1.23	1.22	1.26	0.02	-3.61
West	Denmark	1.30	1.32	1.32	1.30	1.32	1.28	1.29	1.28	1.30	0.03	-3.91
West	Finland	1.37	1.35	1.31	1.29	1.28	1.20	1.18	1.25	1.28	0.05	-10.01
West	France	1.10	1.07	1.09	1.09	1.08	1.06	1.05	1.04	1.07	0.02	-4.98
West	Germany	1.35	1.30	1.30	1.26	1.25	1.23	1.24	1.25	1.27	0.03	-8.49
West	Ireland	1.45	1.46	1.46	1.50	1.36	1.23	1.23	1.21	1.36	0.10	-16.19
West	Italy	1.17	1.13	1.12	1.13	1.11	1.10	1.11	1.13	1.13	0.02	-3.37
West	Luxembourg	1.30	1.32	1.32	1.30	1.26	1.21	1.20	1.26	1.28	0.05	-9.35
West	Netherlands	1.26	1.30	1.30	1.30	1.31	1.27	1.22	1.21	1.27	0.03	-3.33
West	Norway	1.23	1.24	1.18	1.22	1.22	1.15	1.18	1.18	1.19	0.03	-5.86
West	Portugal	1.32	1.32	1.30	1.30	1.29	1.30	1.25	1.23	1.28	0.02	-4.43
West	Spain	1.11	1.07	1.08	1.13	1.10	1.13	1.10	1.09	1.10	0.02	-1.05
West	Sweden	1.14	1.14	1.11	1.11	1.13	1.12	1.12	1.14	1.12	0.01	-0.37
West	Switzerland	1.14	1.08	1.07	1.07	1.04	1.04	1.02	1.05	1.06	0.03	-9.43
West	The United Kingdom	1.31	1.32	1.28	1.27	1.23	1.18	1.17	1.12	1.23	0.07	-14.59
Mean of western countries		1.26	1.25	1.24	1.23	1.22	1.18	1.17	1.18	1.21	0.04	-6.85
East	Belarus ^*a*^	1.46	1.67	1.78	1.95	2.09	2.11	2.11	2.19	1.94	0.23	46.42
East	The Czech Republic	1.80	1.64	1.60	1.57	1.59	1.59	1.53	1.58	1.61	0.07	-13.48
East	Estonia ^*a*^	1.64	1.75	1.67	1.67	1.69	1.74	1.73	1.64	1.70	0.05	-0.83
East	Hungary	1.68	1.70	1.69	1.73	1.68	1.76	1.73	1.75	1.71	0.03	3.02
East	Latvia ^*a*^	1.63	1.78	1.74	1.69	1.81	1.93	1.94	1.86	1.80	0.10	12.85
East	Lithuania ^*a*^	1.36	1.52	1.50	1.49	1.59	1.66	1.70	1.74	1.58	0.12	24.70
East	Poland	1.59	1.60	1.61	1.60	1.54	1.54	1.56	1.54	1.57	0.03	-3.36
East	Slovakia	1.67	1.64	1.63	1.66	1.68	1.76	1.77	1.73	1.71	0.06	2.61
East	Slovenia	1.45	1.52	1.44	1.47	1.47	1.45	1.35	1.37	1.44	0.05	-7.45
East	Ukraine ^*a*^	1.57	1.80	1.92	1.91	2.05	2.16	2.15	2.11	1.98	0.18	30.96
Mean of eastern countries		1.58	1.66	1.66	1.67	1.72	1.77	1.76	1.75	1.70	0.09	8.73
Mean countries of the former USSR		1.53	1.70	1.72	1.74	1.85	1.92	1.93	1.91	1.80	0.15	22.13
Mean of the other former communist countries		1.64	1.62	1.59	1.60	1.59	1.62	1.59	1.60	1.61	0.05	-3.78

^a^countries of the former USSR.

**Table 6:** Behaviour of the CMF in Europe countries for women between 0 and 14 ages over the period 1990-2012

East/West	Country	1990	1993	1996	1999	2002	2005	2008	2011	Mean	SD	RV %
West	Austria	0.71	0.71	0.65	0.67	0.63	0.80	0.66	0.69	0.71	0.07	12.55
West	Belgium	0.73	0.71	0.64	0.70	0.72	0.74	0.75	0.80	0.74	0.04	12.88
West	Denmark	0.68	0.62	0.68	0.58	0.71	0.67	0.72	0.80	0.69	0.06	11.18
West	Finland	0.58	0.47	0.53	0.63	0.53	0.77	0.53	0.60	0.57	0.07	17.51
West	France	0.65	0.63	0.60	0.66	0.69	0.67	0.74	0.74	0.68	0.05	19.15
West	Germany	0.67	0.61	0.62	0.65	0.70	0.71	0.69	0.79	0.67	0.05	10.54
West	Ireland	0.69	0.63	0.72	0.90	0.87	0.85	0.74	0.69	0.75	0.10	14.92
West	Italy	0.68	0.76	0.79	0.71	0.71	0.65	0.69	0.68	0.70	0.04	2.65
West	Luxembourg	0.89	0.61	0.80	0.60	0.72	0.75	0.59	0.63	0.70	0.15	-45.87
West	Netherlands	0.68	0.65	0.69	0.78	0.78	0.86	0.77	0.71	0.73	0.07	15.32
West	Norway	0.63	0.56	0.49	0.63	0.74	0.59	0.54	0.52	0.60	0.06	2.68
West	Portugal	1.05	0.98	1.00	0.99	0.97	0.74	0.76	0.65	0.89	0.14	-19.13
West	Spain	0.72	0.70	0.68	0.70	0.70	0.69	0.70	0.72	0.70	0.02	-3.95
West	Sweden	0.56	0.50	0.47	0.47	0.55	0.53	0.56	0.54	0.53	0.05	7.26
West	Switzerland	0.66	0.67	0.60	0.65	0.71	0.69	0.73	0.83	0.71	0.09	24.25
West	The United Kingdom	0.69	0.65	0.70	0.77	0.78	0.86	0.89	0.86	0.79	0.10	24.31
Mean of western countries		0.70	0.65	0.67	0.69	0.72	0.72	0.69	0.70	0.70	0.07	4.72
East	Belarus ^*a*^	1.06	1.18	1.44	1.54	1.43	1.29	0.95	0.99	1.26	0.18	-10.28
East	The Czech Republic	0.88	0.82	0.77	0.74	0.73	0.70	0.58	0.61	0.73	0.09	-27.34
East	Estonia ^*a*^	1.18	1.48	1.30	1.53	0.92	1.27	0.95	0.75	1.20	0.26	-22.25
East	Hungary	1.24	1.16	1.22	1.09	1.19	1.07	1.06	1.09	1.15	0.07	-4.73
East	Latvia ^*a*^	1.24	1.59	1.80	1.88	1.75	1.59	1.16	1.40	1.62	0.21	8.68
East	Lithuania ^*a*^	1.08	1.40	1.34	1.40	1.28	1.21	1.32	1.09	1.29	0.16	-13.25
East	Poland	1.47	1.38	1.30	1.13	1.10	1.14	1.07	0.98	1.18	0.15	-31.93
East	Slovakia	1.00	1.00	1.14	1.18	1.35	1.20	1.12	1.13	1.15	0.12	13.07
East	Slovenia	0.67	0.67	0.51	0.61	0.56	0.87	0.58	0.65	0.67	0.11	-22.98
East	Ukraine ^*a*^	1.18	1.49	1.74	1.78	1.83	1.89	2.05	1.95	1.76	0.22	65.12
Mean of eastern countries		1.10	1.22	1.26	1.29	1.21	1.22	1.08	1.06	1.20	0.16	-3.87
Mean countries of the former USSR		1.15	1.43	1.52	1.63	1.44	1.45	1.29	1.24	1.42	0.21	6.29
Mean of the other former communist countries		1.05	1.00	0.99	0.95	0.99	1.00	0.88	0.89	0.98	0.11	-14.96

^a^countries of the former USSR

**Table 7:** Behaviour of the CMF in Europe countries for women between 15 and 64 ages over the period 1990-2012

East/West	Country	1990	1993	1996	1999	2002	2005	2008	2011	Mean	SD	RV%
West	Austria	0.54	0.53	0.50	0.51	0.48	0.48	0.47	0.53	0.50	0.02	-2.75
West	Belgium	0.54	0.51	0.51	0.53	0.55	0.55	0.57	0.62	0.55	0.03	12.86
West	Denmark	0.76	0.73	0.71	0.71	0.67	0.64	0.64	0.64	0.68	0.04	-15.61
West	Finland	0.52	0.49	0.45	0.48	0.50	0.54	0.52	0.53	0.50	0.03	2.82
West	Fran e	0.49	0.48	0.47	0.48	0.48	0.48	0.49	0.53	0.49	0.02	9.19
West	Germany	0.58	0.54	0.53	0.51	0.51	0.50	0.52	0.58	0.53	0.02	-1.81
West	Ireland	0.63	0.58	0.54	0.58	0.53	0.52	0.54	0.56	0.56	0.03	-11.10
West	Italy	0.46	0.43	0.42	0.41	0.41	0.39	0.40	0.43	0.42	0.02	-4.52
West	Luxembourg	0.63	0.55	0.56	0.52	0.55	0.49	0.50	0.57	0.55	0.04	-15.83
West	Netherlands	0.50	0.51	0.51	0.55	0.57	0.54	0.55	0.58	0.54	0.03	17.65
West	Norway	0.50	0.47	0.48	0.50	0.51	0.50	0.48	0.52	0.49	0.02	-0.05
West	Portugal	0.57	0.56	0.53	0.53	0.52	0.50	0.47	0.50	0.52	0.03	-14.04
West	Spain	0.43	0.40	0.39	0.38	0.39	0.38	0.39	0.41	0.40	0.01	-4.59
West	Sweden	0.48	0.45	0.44	0.46	0.45	0.46	0.45	0.47	0.46	0.01	-0.03
West	Switzerland	0.46	0.44	0.42	0.42	0.43	0.41	0.41	0.43	0.43	0.01	-4.20
West	The United Kingdom	0.62	0.58	0.55	0.57	0.56	0.55	0.57	0.59	0.57	0.02	-4.62
Mean of western countries		0.54	0.52	0.50	0.51	0.51	0.50	0.50	0.53	0.51	0.02	-3.03
East	Belarus^a^	0.79	0.91	0.98	1.11	1.18	1.17	1.10	1.24	1.05	0.13	34.99
East	The Czech Republic	0.74	0.68	0.63	0.63	0.63	0.64	0.63	0.65	0.65	0.03	-11.69
East	Estonia^a^	0.83	0.94	0.87	0.94	0.90	0.83	0.80	0.73	0.87	0.09	-11.61
East	Hungary	0.99	1.03	0.92	0.98	0.93	0.94	0.95	1.02	0.97	0.03	0.68
East	Latvia^a^	0.87	1.07	0.98	1.02	0.98	1.02	1.00	1.00	1.02	0.09	11.19
East	Lithuania^a^	0.79	0.90	0.90	0.84	0.86	0.96	1.08	1.00	0.92	0.09	22.75
East	Poland	0.77	0.72	0.70	0.73	0.68	0.71	0.74	0.76	0.73	0.03	0.55
East	Slovakia	0.79	0.72	0.67	0.72	0.70	0.71	0.71	0.75	0.72	0.03	-3.65
East	Slovenia	0.60	0.66	0.61	0.62	0.57	0.56	0.51	0.55	0.58	0.04	-6.42
East	Ukraine^a^	0.83	0.97	1.12	1.12	1.19	1.34	1.36	1.19	1.15	0.15	45.99
Mean of eastern countries		0.80	0.86	0.84	0.87	0.86	0.89	0.89	0.89	0.87	0.07	8.74
Mean countries of the former USSR		0.82	0.96	0.97	1.00	1.02	1.06	1.07	1.03	1.00	0.11	20.47
Mean of the other former communist countries		0.78	0.76	0.71	0.74	0.70	0.71	0.71	0.74	0.73	0.03	-3.68

^a^countries of the former USSR

**Table 8:** Behaviour of the CMF in Europe countries for women between 65 and 110+ ages over the period 1990-2012

East/West	Country	1990	1993	1996	1999	2002	2005	2008	2011	Mean	SD	RV%
West	Austria	0.84	0.81	0.81	0.79	0.78	0.77	0.77	0.78	0.79	0.02	-3.54
West	Belgium	0.76	0.76	0.75	0.75	0.78	0.77	0.78	0.79	0.77	0.02	5.95
West	Denmark	0.81	0.85	0.86	0.87	0.91	0.87	0.89	0.89	0.87	0.03	8.32
West	Finland	0.85	0.86	0.81	0.79	0.80	0.73	0.75	0.77	0.79	0.04	-8.62
West	France	0.63	0.61	0.61	0.61	0.62	0.60	0.60	0.61	0.61	0.01	-0.64
West	Germany	0.87	0.82	0.81	0.78	0.79	0.79	0.81	0.83	0.81	0.02	-4.24
West	Ireland	0.91	0.93	0.93	0.96	0.89	0.82	0.83	0.83	0.89	0.05	-9.51
West	Italy	0.73	0.70	0.68	0.69	0.67	0.67	0.69	0.71	0.69	0.02	-0.96
West	Luxembourg	0.83	0.82	0.80	0.80	0.78	0.78	0.79	0.80	0.81	0.03	-2.20
West	Netherlands	0.72	0.75	0.76	0.79	0.82	0.79	0.80	0.81	0.78	0.03	14.34
West	Norway	0.75	0.76	0.72	0.75	0.78	0.72	0.76	0.78	0.75	0.02	6.11
West	Portugal	0.89	0.86	0.85	0.85	0.82	0.83	0.80	0.79	0.83	0.03	-9.24
West	Spain	0.71	0.67	0.66	0.69	0.66	0.68	0.67	0.66	0.67	0.01	-4.74
West	Sweden	0.71	0.72	0.70	0.71	0.75	0.73	0.76	0.78	0.73	0.03	11.88
West	Switzerland	0.68	0.64	0.65	0.66	0.66	0.65	0.67	0.69	0.66	0.02	3.38
West	The United Kingdom	0.81	0.83	0.82	0.84	0.83	0.82	0.83	0.80	0.82	0.01	1.37
Mean of western countries		0.78	0.77	0.76	0.77	0.77	0.75	0.76	0.77	0.77	0.02	0.06
East	Belarus^a^	0.97	1.10	1.16	1.26	1.32	1.31	1.29	1.31	1.22	0.11	30.80
East	The Czech Republic	1.18	1.10	1.08	1.08	1.08	1.09	1.04	1.08	1.09	0.03	-9.52
East	Estonia^a^	1.07	1.12	1.05	1.02	1.05	1.01	1.00	0.96	1.04	0.05	-12.85
East	Hungary	1.14	1.13	1.15	1.16	1.10	1.15	1.12	1.17	1.14	0.02	2.72
East	Latvia^a^	1.08	1.10	1.07	1.09	1.13	1.17	1.16	1.12	1.13	0.04	6.18
East	Lithuania^a^	0.92	1.00	0.98	0.97	1.00	1.04	1.07	1.07	1.01	0.05	14.80
East	Poland	1.03	1.06	1.06	1.03	0.97	0.97	0.98	0.98	1.01	0.04	-5.94
East	Slovakia	1.11	1.08	1.09	1.11	1.12	1.17	1.17	1.19	1.14	0.04	5.56
East	Slovenia	0.95	0.97	0.91	0.90	0.87	0.89	0.85	0.86	0.90	0.05	-9.86
East	Ukraine^a^	1.08	1.24	1.29	1.30	1.37	1.43	1.44	1.44	1.34	0.11	30.02
Mean of eastern countries		1.06	1.09	1.08	1.09	1.10	1.12	1.11	1.12	1.10	0.05	4.89
Mean countries of the former USSR		1.03	1.11	1.11	1.13	1.17	1.19	1.19	1.18	1.15	0.08	13.45
Mean of the other former communist countries		1.08	1.07	1.06	1.06	1.03	1.05	1.03	1.05	1.06	0.04	-3.22

^a^countries of the former USSR

On average, the CMF of eastern countries is high, widening the gap between these countries and the western countries. This gap is higher for men in childhood (0-14) and adulthood (15-64) than women. This means a lower difference for women (Tables 6 and 7), with respect to men (Tables 3 and 4). With respect to the variability of the CMF, the same trend was found. The standard deviation is higher in eastern than western countries, and is especially high for men in childhood and adulthood. This means a lower difference for women (Tables 6 and 7) respect to men (Tables 3 and 4), again.

Focusing on the eastern countries, especially Belarus, Estonia, Latvia, Lithuania and Ukraine (countries of the former USSR) have a mean CMF higher than the rest of the eastern countries. The mean of the CMF is higher for men childhood and adulthood than women. This means a lower difference for women (Tables 6 and 7), with respect to men (Tables 3 and 4), again. Specifically, from 1990 until 1994 an increase in the CMF’s mean for countries of the former USSR was detected for both sexes. It is important to highlight that the mean CMF of countries of the former USSR, especially for men in adulthood, is higher than the rest of Europe. With respect to the variability of the CMF, the same trend was found. The standard deviation is higher in eastern than western countries, and is especially high for men in childhood and adulthood (Tables 3 and 4) compared to women (Tables 6 and 7).

For ages above 64 years, Tables 5 and 8 show higher a mean CMF for men than women both in eastern and western countries. In particular, the mean of the CMF is higher for the countries of the former USSR than the rest of the eastern countries.

With respect to Eastern Europe, it is important to highlight Slovenia as the country with the lowest CMF in Eastern Europe for all age groups, considered years and both sexes (Tables 3 and 8). In the same way, Ukraine as the country with the highest average of CMF of Eastern Europe for all considered years and all age groups and both sexes (Tables 3 and 8). With respect to Western Europe, there is no country that has a CMF higher or lower than the mean of western countries for all years, age groups and both sexes.

Finally, it is noteworthy that Portugal and Luxembourg are the western countries that have the most severely decreased CMF in childhood as shown by the relative variation of -39.27 and -30.03% respectively in Table 3 for men and for women -19.13 and -45.87% in Table 6.

**Fig. 1:**
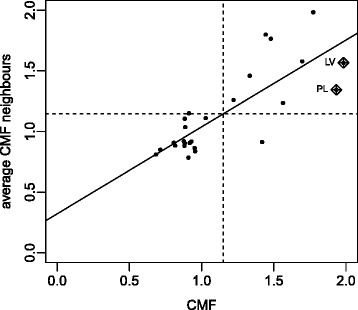
Moran’s Scatterplot for men between 0 and 14 years of age and year 1990 in Europe

**Fig. 2:**
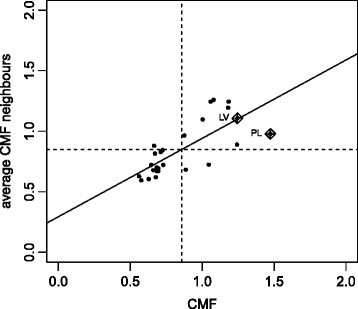
Moran’s Scatterplot for women between 0 and 14 years of age and year 1990 in Europe

After the descriptive quantification of the CMF and in order to test the significance of mortality dependence on space, the null hypothesis of no spatial correlation was tested where the *p*-value is obtained using asymptotic distribution or by means of the Monte-Carlo test [5]. The result of the Moran and Monte-Carlo tests and Robust Index are shown in Table 9 which includes for each age group and years 1990 and 2012 the value of the observed Moran’s I and Robust Index with the *p*-value of the Moran (M) and Monte-Carlo (MC) test and in the last column the *p*-value of Robust Index (RI) for men and women respectively. The *p*-values obtained for all years, age groups and both sexes are significant (*p*-values <0.05), indicating that there is a spatial dependence between the mortality of the countries in the observed period. In addition, the Moran’s Scatterplot shown in the work of Aselin (1993) were obtained for each year, sex and age group considered. Figure 1 represents the Moran’s Scatterplot for men between 0 and 14 years of age and year 1990 in Europe and Fig. 2 the same plot for women. No outliers were observed on Moran’s Scatterplot obtained for each year, sex and age group.

**Table 9:** Values of the Global Moran’s I, Robust Index and *p*-values for men and women

Age group	Year	*G* *M*_*g*,*t*,*s*_		*R* *I*_*g*,*t*,*s*_		*p*-value M		*p*-value MC		*p*-value RI	
		Male	Female	Male	Female	Male	Female	Male	Female	Male	Female
0-14	1990	0.72	0.65	0.72	0.71	0.00	0.00	0.00	0.00	0.00	0.00
0-14	2012	0.36	0.34	0.50	0.50	0.00	0.00	0.00	0.00	0.00	0.00
15-64	1990	0.79	0.62	0.78	0.67	0.00	0.00	0.00	0.00	0.00	0.00
15-64	2012	0.79	0.69	0.79	0.74	0.00	0.00	0.00	0.00	0.00	0.00
65-110+	1990	0.57	0.63	0.61	0.65	0.00	0.00	0.00	0.00	0.00	0.00
65-110+	2012	0.78	0.70	0.81	0.74	0.00	0.00	0.00	0.00	0.00	0.00

As stated in “Local Moran’s Index” section, LISA values permit the detection of a significant cluster of countries. Figures 3 and 4,^1^ show significant clusters of different European countries, identifying the centre and neighbours. The centre cluster can be unique and represents the country located in the middle of the cluster. When several centre clusters appear inside a single cluster, it means that there are several clusters belonging to bordering countries which form a single macrocluster. Figure 1 shows the significant clusters in Europe for men, between 65-110+ ages for years 1990 and 2012 and Fig. 2 for women. Depending on the year, sex and age group considered, the countries forming the different clusters and its centre cluster vary but comparing all maps made, two significant clusters are observed, especially for people older than 64: a cluster of high CMF (HH) consisting of Eastern European countries (Poland, Lithuania, Latvia, Estonia, Ukraine, Belarus, Slovakia and Hungary) and another cluster of low CMF (LL) consisting of Western European countries (The United Kingdom, Austria, Spain, Italy, France, Switzerland, Germany, Luxembourg and Belgium), as the Local Moran’s I values indicates. Non-significant values of the Local Moran’s I therefore identify countries which do not belong to any cluster (The Czech Republic, Portugal, Denmark, Finland, Ireland, The Netherlands, Norway, Slovenia and Sweden). In this case, it is necessary to emphasize two facts: the first is that in some occasions Austria belongs to the cluster consisting of Eastern European countries. This is due to the fact that Austria has a common border with Slovakia and Hungary, countries that occasionally belong to the centre cluster. The second event is that there are several clusters of type HH (high CMF) which form a unique macrocluster HH. For this reason in the cluster HH several centre clusters are observed. These centre clusters differ over the same period, moving from the west to the east of Europe. In the clusters of type LL (low CMF) one or two centre clusters are observed depending on the age group, year and sex considered. Sometimes the centre is unique (France) and sometimes two centres are observed (France and Switzerland) forming a single LL macrocluster.

In contrast, for ages below or equal to 64 only one significant cluster of type HH formed by countries in Eastern Europe (Poland, Lithuania, Latvia, Estonia, Ukraine, Belarus, Slovakia and Hungary) is observed, whereas the values of the Local Moran’s Index of other countries are non significant.

Next the space-time interaction was studied as described in “Spatial Markov” subsection. For this purpose, the estimates of transition probability matrices were calculated for each age group and sex for the period 1990-2012. These probabilities are estimated assuming temporal homogeneity throughout the corresponding observed frequencies. Table 10 shows the values of the *χ*^2^ test for both sexes and each age group during the period 1990-2012. Very high values of the *χ*^2^ test mean that the *p*-values associated with the test are very significant and conclude that the space-time interaction is significant, i.e. the CMF in a country and in its surroundings evolve jointly during the period 1990-2012 for all age groups and both sexes. In particular, the space-time interaction for men in adulthood is the most significant. The results of Table 10 confirm our findings in Figs. 1 and 2 where clusters vary over time depending on the age group and sex considered. In this case, it can be said that mortality in EU not only depends on differences in the health systems, which are a subject to national discretion, but also on supra-national developments.

**Table 10:** Values of the *χ*^2^ test for men and women

Age group	***χ*** ^***2***^		*p*-valor
	Male	Female	
0-14	61.63	82.39	0.00
15-64	158.02	85.49	0.00
65-110+	29.78	80.75	0.00

## Conclusions

Though the mortality gap between eastern and western countries is growing according to our results, this has not attracted as much attention recently as it deserves [22, 42]. There are clear differences in mortality between the east and west of the EU that are more important than the traditional south-north division, with a significant disadvantage for Eastern Europe [12]. In particular, men are heavily burdened by high mortality, especially in adulthood. Differences between eastern and western countries show very large preventable burdens of ischemic heart disease, strokes, other heart diseases, smoking related cancers, injuries and alcohol related mortality [8].

This paper quantifies the dynamics of mortality in Europe and in turn, detect significant differences in mortality between countries using measurements and techniques such as: CMF, Global Moran’s index, Local Moran’s index and Spatial Markov. Specifically, the CMF provides a suitable statistic for comparison of mortality, standardised by the direct method, for each country by sex and age group. The CMF was used properly and not SMR, which is the most commonly used technique. The two indexes of spatial autocorrelation are widely used in econometrics, epidemiology and demography, both being static indices in the sense that they measure spatial autocorrelation at a moment in time [33, 34]. The Spatial Markov technique is used to test the spatio-temporal significance effect of the interaction between time and space in the analysis of mortality in different countries.

Firstly, the main conclusions about the results to quantify the dynamics of mortality in Europe detailed in “Results” section are described below.

The gap between Eastern and Western Europe is widening because, considering the average and standard deviation, the CMF of eastern countries is higher than western countries. This gap is greater for men in childhood and adulthood than for women.

Focusing on the eastern countries from the former USSR, they have a mean and standard deviation of CMF higher than the rest of the eastern countries for men in childhood and adulthood, again. To highlight the high mean CMF in countries of former USSR for men in adulthood compared to the rest of Europe. Our results for a more recent period are consistent with those obtained in papers such as Meslé et al. and Bonneux et al. [8, 26], which show that in Eastern European countries, and especially countries of the former USSR, excess mortality in adult ages in men is spectacularly high. Specifically, from 1990 until 1994 an increase in the mean CMF in countries of former USSR was detected. This fact is associated with the collapse of the Soviet Union, which between 1990 and 1994 caused life expectancy in these countries to diminish [22].

Portugal suffered the largest male and female decline of CMF in infants for 1990-2012. These gains are explained by massive reductions in the infant mortality rate (IMR) as in 1970, Portugal had the highest IMR (50 per 1000 live births) of any of the European countries [22]. With respect to Luxembourg, it also suffered a decrease of CMF in infants for 1990-2012. This decrease is described by the United Nations Children’s Fund which announced that Luxembourg in 2012 reached an under five mortality rate (U5MR) of 2 deaths per 1.000 live births with the average annual rate of reduction of 6.2% in 1990-2012 [41].

Slovenia is the Eastern country with below average CMF for all age groups, considering years and both sexes. Trnka et al. [40] point out that between 1994-1996 mass primary vaccination and general revaccination were extremely common in eastern countries where the prevalence of tuberculosis was high. Slovenia was the only country belonging to Central and Eastern Europe that adhered to World Health Organization (WHO) guidelines, where revaccination and tuberculin skin tests were not applied. Similarity, Ukraine has an above average CMF for eastern countries for all considered years, both sexes and all age groups. This occurred because while recent trends in the health situation in central European countries are quite favourable, the future for countries of the former USSR such as Russia and the Ukraine is negative, they show no signs of improvement, and mortality has continued to increase [25]. With respect to Western Europe, there is no country that has a CMF higher or lower than the mean for western countries for all years and age groups.

Next, the most important conclusions to detect significant differences in mortality applying spatio-temporal methodology between countries are detailed.

A spatial correlation in the mortality of the 26 studied European countries was found, as the Global and Local Moran’s I values indicate.

Different significant clusters depending on the year, sex and age group considered were observed but mainly for ages older than 64, two significant clusters were obtained: a cluster of countries with high values of CMF surrounded by neighbours also with high values of CMF formed by Eastern European countries (Poland, Lithuania, Latvia, Estonia, Ukraine, Belarus, Slovakia and Hungary) and another cluster of countries with low values for CMF surrounded by neighbours also with low values of CMF composed of Western European countries (The United Kingdom, Austria, Spain, Italy, France, Switzerland, Germany, Luxembourg and Belgium). Non-significant values of the Local Moran’s I identify countries which do not belong to any cluster (The Czech Republic, Portugal, Denmark, Finland, Ireland, The Netherlands, Norway, Slovenia and Sweden). In contrast, for ages below or equal to 64 only one significant cluster of countries with high values of CMF surrounded by neighbours also with high values of CMF formed of Eastern European countries (Poland, Lithuania, Latvia, Estonia, Ukraine, Belarus, Slovakia and Hungary) was observed, whereas the values of the Local Moran’s Index for other countries were non significant, not belonging to any cluster.

Finally, the Spatial Markov method confirms the existence of a space-time interaction, meaning that there is spatio-temporal dependence between the 26 studied European countries during the period 1990-2012. For this reason, the obtained clusters vary over time depending on the age group and sex considered. In particular, the space-time interaction for adult men is the most significant. In this case, it can be said that mortality in EU not only depends on differences in the health systems, which are a subject to national discretion, but also on supra-national developments.

In relation to the work of other authors, we should highlight two distinctive features of the methodology presented here which is the possibility of considering neighbour relations between countries, the element of time and their interaction. There are previous studies that address the differences in life expectancy at birth in Europe. Meslé and Vallin [26] who performed a hierarchical cluster analysis of age-specific mortality patterns by sex in years 1965 and 1995. Leon [22] studied the trends in life expectancy at birth in Europe and Japan by sex using a graph that shows the temporal evolution of this variable during 1970-2008. Shaw et al. [37] analysed geographical patterns in mortality, for 160 regions of 15 European countries in years 1990 and 1994. In that last paper, the mortality was quantified by SMR and was presented for different causes of death by means of maps without distinguishing between men and women separately. All these authors study differences of mortality in Europe, but none of them quantify the differences in mortality by age and sex, checking in turn that they are significant using a spatio-temporal methodology. Therefore, our study is complementary to those other techniques.

In short, this paper proposes novel statistical tools which provide a clear framework for the successful implementation of development public policies and help the UE to meet the challenge of rethinking its social model (Social Security and health care) and make it sustainable in the medium term [3].

Let us highlight, finally, that the natural extension of this paper is fitting a spatio-temporal panel data model with covariates to these mortality data. This model studies the space and temporal dependence of data and to analyzes the effect of covariates on CMF. This can be considered a future project.

## Abbreviations

AT: Austria
BE: Belgium
BY: Belarus
CH: Switzerland
CMF: Comparative mortality figure
CZ: Czech Republic
DE: Germany
DK: Denmark
EE: Estonia
ES: Spain
EU: European Union
FI: Finland
FR: France
H: Higher
HU: Hungary
HMD: Human mortality database
IE: Ireland
IMR: Infant mortality rate
IT: Italy
L: Lower
LISA: Local indicators of spatial association LT: Lithuania
LU: Luxembourg
LV: Latvia
M: Moran’s test
MC: Monte-Carlo test
NL: Netherlands
NO: Norway
PL: Poland
PT: Portugal
RI: Robust Index
RV%: Relative variation in percentage
SD: Standard deviation
SE: Sweden
SI: Slovenia
SMR: Standardised mortality ratio
SK: Slovakia
UA: Ukraine
USSR: Countries of the former Union of Soviet Socialist Republics
UK: United Kingdom
U5MR: Under five mortality rate
WHO: World Health Organization

## Footnotes

1 The shapefile maps used in this publication are based on the following source: ⒸEuroGeographics for the administrative boundaries. Particularly, the world shapefile maps of scale 1:20 Million corresponding to period 2013 were used. http://ec.europa.eu/eurostat/cache/GISCO/geodatafiles/NUTS_2013_20M.zip
